# The Properties of Water and their Applications for Training

**DOI:** 10.2478/hukin-2014-0129

**Published:** 2014-12-30

**Authors:** Lorena Torres-Ronda, Xavi Schelling i del Alcázar

**Affiliations:** 1 National Institute of Physical Education of Catalonia (INEFC), Lérida, Spain; 2 Complex Systems in the Sport Research Group, Barcelona, Spain.

**Keywords:** aquatic training, rehabilitation, recovery

## Abstract

The biological effects of immersion in water, which are related to the fundamental principles of hydrodynamics, may be beneficial in certain training contexts. The effects and physical properties of water, such as density, hydrostatic pressure and buoyancy are highly useful resources for training, when used as a counterbalance to gravity, resistance, a compressor and a thermal conductor. Not only does the aquatic medium enable a wider range of activities to be used in a context of low joint impact, but it also constitutes a useful tool in relation to sports rehabilitation, since it allows the athlete to return to training earlier or to continue with high-intensity exercise while ensuring both low joint impact and greater comfort for the individual concerned. Moreover, this medium enables the stimulation of metabolic and neuromuscular systems, followed by their corresponding physiological adaptations allowing both to maintain and improve athletic performance. Hydrotherapy can also play a beneficial role in an athlete’s recovery, helping to prevent as well as treat muscle damage and soreness following exercise.

## Introduction

Upon entering water the human body encounters a medium for which it is not designed, one in which locomotion is of limited efficiency. However, the biological effects of immersion in water, which are related to the fundamental principles of hydrodynamics, may be beneficial in certain training contexts. Understanding these effects and the physical properties of water, such as density, hydrostatic pressure, and buoyancy, may therefore help coaches and trainers to design not only fitness programs but also protocols for post-exercise recovery and/or sports rehabilitation.

Training in the aquatic medium does not refer solely to different styles of swimming (crawl, backstroke, breaststroke, or butterfly), which also requires a certain degree of technical skills. On the contrary, this medium offers numerous other possibilities, since a person can, under different conditions, perform typically terrestrial activities such as walking, jogging, running, or jumping, not to mention other specific movements associated with sport. This enables the stimulation of metabolic and neuromuscular systems, followed by their corresponding physiological adaptations. In terms of sports rehabilitation, the advantage of this medium is that it allows activities or exercises to be introduced in the early stages of re-adaptation, thereby activating the functions of the musculoskeletal and cardiovascular systems while maintaining a low risk of injury.

In light of the above, the aim of this paper was to propose ways of using this medium both to optimize fitness and to promote recovery and rehabilitation among athletes. Preceding each section, a brief description on the physical properties of water, that need to be considered when employing aquatic-based activities, is presented. As this is not a systematic review, only those studies which are regarded as relevant to these goals are considered. The recommendations put forward are aimed primarily at intermediate to professional level athletes, and no recommendations or references are made in relation to special populations (e.g., sedentary individuals, clinical cases, or pregnant women, etc.; for a review, see Becker (2009)).

## Water as a Counterbalance to Gravity

Density (ϱ) is the ratio between the mass of a substance (kg) and the space it occupies (m^3^). In case of a fluid, the greater its density the greater the force it exerts on objects that float or are immersed in it. The density (specific weight) of water at 1 atmosphere pressure and 4 °C is 1000 kg/m^3^ for freshwater and 1027 kg/m^3^ in case of saltwater; the latter value is higher because the dissolved salt (sodium chloride) content of saltwater means that it contains more molecules of solute in the same volume. The density of liquid water is highly stable and varies little with changes in temperature and pressure. Due to its density, water acts as a counterbalance to the effects of gravity, such that a body submerged in it will float (an object will float in a fluid provided that the number of particles it contains is less than the number of displaced fluid particles). The mean density of the human body is 950 kg/m^3^, although the figure obviously varies depending on sex (generally greater in men), somatotype (an individual with greater muscle weight tends to have a specific gravity greater than 1, whereas this tends to be less than 1 in someone with a higher fat percentage), and age, etc. (Becker, 2009). The relationship between the density of water and that of the human body (the latter is lower) means that an individual can float in water (Archimedes’ principle), an effect that is easier to achieve in saltwater than in freshwater, and also in cold as opposed to warm water.

Hence, density is related to buoyancy. The Archimedes’ principle states that when a body is partially or totally immersed in a fluid it is buoyed up by a force equal to the weight (volume) of the fluid that is displaced by the body. In other words, as the body is submerged it displaces water, which in turn produces the buoyancy force. However, the force of gravity also acts on this body, which means that if the body is to remain in static equilibrium (neutral buoyancy) these two forces must counterbalance one another, that is, the buoyant force must be equal to the weight (∑F=0) if the body is to remain suspended in the fluid. Buoyancy will be positive when a body tends to rise within the fluid and negative when it tends to sink. Therefore, a body’s buoyancy can change if its weight and/or volume are modified.

### Applications for Training

The fact that water acts as a counterbalance to the force of gravity means that as a medium it has a reduced impact on joints. This property can be highly useful in processes of rehabilitation and recovery, as well as for overweight people or those who suffer joint pain. The percentage of body weight that is off-loaded, or the magnitude of forces exerted, will depend on several factors: 1) the depth of immersion, 2) the performance speed, and 3) gender (Haupenthal et al., 2013; Roesler et al., 2006). A person whose body is immersed to the symphysis pubis will off-load approximately 40% of his or her body weight, and when immersed further to the umbilicus, around 50%. Xiphoid immersion off-loads body weight by 60% or more, while further immersion to the shoulders will off-load around 85%, depending on the position of the arms (Alberton et al., 2011; Becker, 2009; Killgore, 2012).

Several studies have analyzed locomotion (walking, jogging, or running) during water immersion (Alberton et al., 2011; Killgore, 2012). The factors to consider are principally as follows: 1) whether the exercise is static or involves horizontal or vertical movement; 2) the performance speed: submaximal or maximal; and 3) the depth of immersion. When comparing exercise performed in the aquatic and dry land environment, research has found that when an exercise involves a horizontal movement at submaximal speeds (e.g., aquatic treadmill, walking or jogging), at a depth shallow immersion, oxygen uptake (VO_2_) and muscle (electromyographic; EMG) activity are greater than when performing the same exercise at the same speed on land (Becker, 2009). Conversely, when the exercise is static or involves a vertical movement the metabolic cost, the cardiovascular responses and neuromuscular activity are reduced in comparison with the same exercise on land, this being due to the reduction in body weight produced by the effect of buoyancy (Alberton et al., 2011; Reilly et al., 2003). Recently, Alberton et al. (2011) reported significant differences (p<0.001) in VO_2_ and %VO_2peak_ between stationary running performed in water and on dry land (∼10 to 20 ml·kg^−1^·min^−1^ and ∼20 to 40%, in water, ∼20 to 30 ml·kg^−1^·min^−1^ and ∼40 to 60% on land, for VO_2_ and %VO_2_peak, respectively), as well as EMG variables at submaximal cadences elicit higher values (p<0.01) for dry land compared with the aquatic environment, supporting previous findings. The effects are different, however, when the exercise (static, horizontal, or vertical) is performed at high or maximal speeds, both the metabolic cost and neuromuscular activity are very similar to or even greater than would be the case on land, this being due to the fact that the buoyancy effect is counteracted by water resistance (Alberton et al., 2011; Becker, 2009; Jones et al., 2011).

Several studies have analyzed the effect of water-based exercise training, both deep- and shallow levels. The results of these investigations suggested improvements in maximal aerobic capacity (VO_2_peak) ranging from ∼12 to 40% in sedentary or a lower level of fitness population, reelecting improved aerobic capacity in untrained subjects (Jones et al., 2011), while it helps in a rehabilitation process in competitive athletes (Alberton et al., 2011; Becker, 2009; Jones et al., 2011; Reilly et al., 2003; Thein and Brody, 1998). Furthermore, deep water jogging-, running-, cross- training have proven to be effective training to maintain fitness (running performance, VO_2max_, heart rate, lactate threshold, among other parameters) in trained athletes (Bushman et al., 1997; Darby and Yaekle, 2000; Gatti et al., 1979; Wilber et al., 1996). It should be kept in mind that athletes need to use similar intensity to that used during land-based training to ensure an effective workout, and also a proper technique is essential (Haff, 2008). The potential of these results is highly valuable for athletes, since performing such training would allow to maintain fitness while diminishing the risk of injury.

With regard to the depth of immersion, the greater it is, the weaker will be the ground reaction force (GRF) in the vertical plane. However, the GRF in the anterior-posterior plane will be greater as the depth of immersion increases, as more force is required to move the body forward against water resistance. The GRF in the anterior-posterior plane also depends on the speed of movement, such that the greater the running speed the greater the GRF (Barela et al., 2006; Fontana et al., 2011; Haupenthal et al., 2013; Haupenthal et al., 2010; Roesler et al., 2006).

Although water is a medium that reduces impact and load in the vertical plane, few studies have analyzed its effects on training involving jumps and multi-jumps. Performance of high-intensity explosive exercise often produces muscle damage, due mainly to the eccentric component of the muscle action, as well as to the joint load (ligaments and tendons), which constitutes a potential cause of lesion (LaStayo et al., 2003). In water, buoyancy force controls the downward movement of the body, thus generating higher concentric forces while reducing impact forces and joint loading, from ∼45% and ∼60% in peak GRF during single- and double-leg squat jumps in water (varying depending on water depth, jumping and landing technique, among other factors) (Colado et al., 2010; Donoghue et al., 2011; Thein and Brody, 1998). Moreover, studies that have compared plyometric training in the aquatic medium (at water depth of chest, waist and knee) with that performed on dry land reported no differences in terms of maximum force or muscle power achieved, nor in the speed or height of jumps, for both male and female athletes (Donoghue et al., 2011; Martel et al., 2005; Robinson et al., 2004; Stemm and Jacobson, 2007b). Furthermore, the aquatic medium was found to be associated with a reduction in vertical impact forces, as well as in inflammation and perceived pain (Arazi and Asadi, 2011; Arazi et al., 2012; Haupenthal et al., 2011; Martel et al., 2005; Miller et al., 2002; Robinson et al., 2004; Stemm and Jacobson, 2007a). These results support the use of aquatic plyometrics, as it can represent a progression before reintroducing full-effort land-based plyometrics (allowing to reestablish appropriate movement patterns in a non-impact medium), and lead to improved performance while reducing both musculoskeletal stress and perceived post-exercise discomfort.

In order to take advantage of this property of water and off-load body weight, thereby reducing the impact on joints, it is obviously necessary for part of the body to be immersed. As noted above, the intensity of the effect will vary depending on the depth of immersion and the speed at which the exercise is performed. One way of controlling the depth of immersion and the degree of off-loading would be to use a swimming pool with a sloping floor. Then, one of the modulators in the progression of training would be the depth. There are also hydrotherapy treadmills, cabins usually with glass walls, in which water height and speed of the treadmill are controlled and altered when required. The potential drawback of this equipment is the limitation of activities that can be performed, reduced to walking, jogging or running. However, if such a pool or equipment is not available the depth of immersion can be altered using some kind of a flotation device. A wide range of flotation aids is available, including pull buoys, hand floats, pool noodles, flotation belts, and inflatable vests. This type of equipment allows to change the center and level floatation and, thereby, athletes who do not have a mastery of aquatic environment can perform typically terrestrial activities in this medium.

In terms of the program content, attention must be paid to the usual variables that are manipulated when designing training sessions, for example, the duration and intensity of stimuli, the density (i.e., the work-to-rest ratio), the number of sets and repetitions, and the periodization schedule, all of which will depend on the training objective.

## Water as Resistance

Resistance offered by water is greater than that experienced during locomotion on dry land (Dowzer et al., 1998). This greater resistance of water compared with that of air is due not only to its density, but also to its dynamic viscosity. The latter refers to the magnitude of the internal friction associated with a given fluid, in other words, its resistance to flow. When the whole body (or one of its extremities) moves through water it is subjected to three types of resistance: 1) shape resistance, 2) wave resistance, and 3) friction. Shape resistance is caused by the fact that movement through water produces an area of high pressure in front of the individual, in the direction of movement, and a low pressure region behind, where the laminar flow of the water is replaced by the turbulent flow. This resistance increases in line with the force that is exerted against the water, or to put it another way, the greater the surface area that “collides” with the water the greater the resistance. This is why, when swimming in a horizontal position, one tries to maintain an optimum body alignment and the maximum possible buoyancy. Wave resistance is caused by the body colliding with the waves that are produced by its progression through the water or by movement near the surface, especially up and down movements of body segments. The final, and perhaps least noteworthy form of resistance, friction, is due to the resistance offered by water immediately upon coming into contact with the body. As in case of shape resistance the amount of friction will depend on the contact surface area, although it is also influenced by the water’s viscosity, the friction coefficient of the person’s skin, his or her hair and swimwear, and swim speed.

### Applications for Training

Given the nature of resistance (force) that is offered by water, the load or resistance that has to be overcome is less when stationary or when moving at a low or moderate speed. However, the load will increase when high-speed movements are made. This offers the possibility of performing high-speed resistance training without overload and in an environment that produces low joint impact.

Various studies have concluded that performing a training program in water can lead to significant improvements not only in strength and muscle power in both the upper and lower extremities, but also in maximum oxygen uptake (VO_2max_), calorie expenditure, respiratory function, flexibility, and body composition (Alberton et al., 2011; Colado et al., 2009; de Souza et al., 2012; Pinto et al., 2014). As mentioned, specific aquatic plyometric training demonstrated increases in vertical jump performance in female volleyball players (Martel et al., 2005). Moreover, Pinto et al. (2014) studied the effect of intra-session exercise order during concurrent training performed in the aquatic environment. Their results showed that individuals who performed resistance training before the aerobic exercise achieved greater maximal dynamic strength adaptations (43 vs. 27%), greater muscle thickness (10 vs. 6%) and elbow flexors (5 vs. 3%) compared to those who performed the exercise in the opposite order. However, there are few references regarding strength gains thought aquatic training within competitive athletes. Surely this is due to the difficulty to achieve enough intensity in the aquatic environment to ensure strength gains. Nevertheless, all this can be highly useful in the context of rehabilitation, recovery, or during transition periods as a way of maintaining the training stimulus (neuromuscular load, musculoskeletal tension, and metabolic/energy systems) through a modified training schedule while decreasing a joint load and reducing muscle soreness. In this regard, and as in case of traditional fitness training (on dry land), it is necessary to consider the aims of training (i.e., geared more towards cardiovascular or neuromuscular aspects), the modulators of load (volume, intensity, recovery time, work-to-rest ratio, etc.), the exercise schedule, and the implications of using open or closed kinetic chain exercises, etc.

When it comes to strength (or resistance) training there are numerous items of equipment (gloves, paddles, fins, drag swimsuits, etc.) that, by increasing the area of “collision”, increase the water’s resistance and, therefore, the force required to move the item through the water. Previous investigations have confirmed greater responses from the HR and VO_2_ with an increasing cadence (resulting in higher activation of the muscles) and with the use of equipment (de Souza et al., 2012; Pinto et al., 2011). Also, items such as elastic bands or medicine balls can be used with the same aims as on dry land.

In summary, this kind of training offers an alternative to land-based workouts and, whether in the context of post-injury recovery or for athletes who have suffered muscle and/or joint overload, enables the continuation of high-intensity exercise (running, jumps, or throws) while ensuring not only low joint impact (since the water’s density and viscosity cushions any movement and allows increased control over it), but also greater comfort for the individual concerned. Note, however, that for the same reasons that the aquatic medium cushions movement in it, the technical performance of some movements (e.g., walking, running, etc.) will also be altered (Alberton et al., 2011).

## Water as a Compressor

When an individual is submerged in water he or she is subjected to *hydrostatic pressure* (P), which is the pressure that water, or any other fluid, exerts on itself. This pressure is directly proportional to the density of the liquid (ϱ), gravity (g), and the depth to which the body is immersed (h) (Wilcock et al., 2006a). This means that the hydrostatic pressure depends neither on the shape or surface area of the recipient or container (e.g., a swimming pool) nor on the volume of liquid, but primarily on the depth of immersion. In other words, the deeper the immersion the greater the pressure that is exerted on the body; an individual who is submerged to a depth of 10 m is subjected to a pressure of 2 bar, which means that the pressure exerted by just 10 m of water is the same as that generated by the entire atmosphere of air at the sea level (Bove, 2002), equivalent to ∼80 mm Hg, slightly higher than normal diastolic blood pressure (Wilcock et al., 2006a).

The pressure that water exerts on a body that is immersed in it has two effects: 1) fluids are driven from the extremities towards the central cavity: blood flows upwards through the venous and lymphatic systems towards the thoracic area, resulting in increased circulation first in the muscles, then in the blood vessels of the abdominal cavity, and finally in those of the chest cavity and heart; and 2) the chest wall is compressed, altering pulmonary function and respiratory dynamics and increasing the work of breathing.

### Applications for Training

Due to hydrostatic pressure the organism’s responses to exercise performed in the aquatic medium are different to those produced during land-based exercise. Exercising in water produces an increase in cardiac output, in the blood flow to muscles, and in the diffusion of metabolic waste products from muscle to blood, as well as a reduction in the time it takes to transport oxygen, nutrients, and hormones to fatigued muscles (Versey et al., 2013; Wilcock et al., 2006a; Wilcock et al., 2006b). Cardiac volume increases by 30–35% with immersion to the neck (Becker, 2009; Meredith-Jones et al., 2011). The pressure exerted by water also leads to a reduction in partial oxygen pressure, thereby increasing the work of breathing by 60% compared with what occurs on dry land and a higher minute ventilation response is needed to produce an oxygen consumption equivalent to that when exercising on land (Meredith-Jones et al., 2011). Lung function is also reduced, since the hydrostatic pressure exerted by water counteracts the action of inspiratory muscles, compressing the abdomen and raising the diaphragm to a position close to full expiration.

As noted earlier, stationary running at sub-maximal speeds in water produces a lower cardiorespiratory and neuromuscular response than would be the case on dry land, regardless of how fit or experienced the subject is (Alberton et al., 2011; Meredith-Jones et al., 2011). The reduction in the maximum heart rate (HR_max_) that occurs in water is due to the increased hydrostatic pressure on the lower part of the extremities, which as we have seen leads to a reduction in the peripheral blood flow and a redistribution of blood towards the chest area. This increases stress on the heart, leading to increased systolic volume and a subsequent reduction in the heart rate. The reduction in VO_2max_ during submaximal exercise is attributed to the fact that fewer muscles are involved, since immersion can alter the processes associated with contraction or trigger mechanisms of inhibition (with a subsequent reduction in neuromuscular activation) (DeMaere and Ruby, 1997; Wilcock et al., 2006a).

Despite the above, some studies suggest that aquatic training, whether in deep or shallow water, can improve the maximal aerobic capacity of unfit subjects and maintain the aerobic fitness of competitive athletes (Meredith-Jones et al., 2011). It may also help to strengthen respiratory muscles, which could be a benefit for certain athletes. A program of water-based exercises can be a positive stimulus for athletes unaccustomed to training in water, as the work will presume an increase in workload demand on the respiratory system and an improvement in strength of the inspiratory muscles has proven to be effective in improving athletic performance (Becker, 2009). Once again, attention must be paid to the depth of immersion, to the duration and intensity of exercise, and to the individual characteristics of each athlete in order to obtain optimal outcomes.

When considering this property in the recovery processes, the effects of hydrostatic pressure can limit the formation of edema, thereby reducing the effect of muscle damage (whether following exercise or due to a lesion) (see section 5 below), and may also help to maintain both oxygen supply to muscles and contractile function. In addition, water immersion has been shown to be effective at improving the elimination of intramuscular metabolic byproducts and enhancing plasma creatinine (PCr) recovery (Wilcock et al., 2006a).

## Water as a Thermal Conductor

Of particular interest among the thermodynamic properties of water is its specific heat or energy efficiency (first law of thermodynamics or the law of energy conservation). Due to its molecular structure, water has a high capacity to retain heat, to maintain a constant temperature, and to transfer heat energy (it is a good thermal conductor). The fact that water is an efficient thermal conductor, combined with its high specific heat, means that it is a very stable medium for retaining heat or cold. Furthermore, given that the heat capacity (i.e., the capacity to store heat) of the human body is less than that of water (0.83 *vs.* 1.00 kcal/kg-°C) the human body will be quicker to reach a new equilibrium than will water. This means that when an individual is submerged in cold or warm water, it will be his or her body that adapts to the water temperature, rather than vice versa.

### Applications for Training

In the context of training, this thermal property of water is used mainly as a way of controlling the edema and inflammation associated with a lesion, of minimizing fatigue and promoting an athlete’s recovery, or in order to optimize performance. The different techniques are usually considered according to the water temperature involved, namely cold- and cool-water immersion or cryotherapy (≤ 20 °C), warm water immersion (≥ 36 °C), contrast baths (alternating cold and warm water), or thermoneutral immersion (21–35 °C).

### Before and during exercise

The most widely used technique in this context is cold water immersion or cryotherapy, especially in hot environments (> 26 °C). The proposed physiological mechanism underlying precooling (i.e., before exercise) is that it reduces heat stress on the thermoregulatory system and, consequently, delays the onset of thermally induced fatigue. Numerous studies have examined the effect of cooling before and during exercise including very recent reviews (systematic analysis and meta-analysis) (Bongers et al., 2014; Ross et al., 2013; Tyler et al., 2013; Wegmann et al., 2012).

Research provides strong support for the positive effects of precooling, specifically its ability to reduce perceived heat stress and improve subsequent athletic performance. Pre-cooling has a larger effect on performance in hot (+6.6%) than in moderate temperatures (+1.4%). Cooling water immersion can lead to a rapid and large reduction of core body temperature and, therefore, can influence performance, in most cases positively, although in few studies, negatively (when cooling induces physiological alterations such as a reduction of more than 1.9°C in body temperature, extreme vasoconstriction and a decrease in muscular temperature (Wegmann et al. 2012). In general, the effect on performance is moderate to large (∼4 to ∼37%), although this varies depending on the type of activity: the effect on performance tends to be less in case of sprints or intermittent sprints, whereas performance in intermediate and, especially, endurance events is improved by precooling. A wide range of protocols (according to the duration of exposure, the temperature of the water, and the body surface area that is exposed) has been used to analyze the effect of cold water immersion on performance: i) whole body: e.g. cool-water (12°C)-short duration (15′); cool- (17°C) to warm-water (25–30 °C)-long duration (30′); warm water (∼24°C)-long duration (60′), ii) lower body: e.g. cool water (14°C)-long duration (20′), and iii) upper body: cool water (14–18°C)-long duration (30′).

The use of cooling strategies during exercise can also have a positive effect on physical performance. Precooling contributes to a higher heat storage capacity, a more efficient heat loss and may attenuate an increase in body temperature, which may prevent a decrease in exercise performance in endurance exercise (Bongers et al., 2014; Tyler et al., 2013). Nonetheless, Stanley et al. (2013) reported that cold water immersion, between high-intensity exercises increased cardiac parasympathetic activity, slowed VO_2_ on-kinetics and reduced muscle O_2_ utilization. Consequently, there was an increase in the anaerobic contribution during subsequent exercises, or from another point of view, cold water immersion has detrimental effects on high-intensity exercise (e.g. in sports that involve intensity actions during competition or when the aim of the training is to increase aerobic capacity) that seems to persist ∼45 min. These results have important practical applications, since it would not be appropriate to use this strategy just before competing or prior to high-intensity events, if they are not separated by at least the time above-mentioned. Despite relatively little is known about the impact of precooling on exercise performance this method seems to be effective.

Although it appears that cold water immersion is not always the most effective method in terms of outcomes (as compared e.g. with a cooled room), it remains a highly practical and efficient approach, particularly in competitive situations. Nonetheless, it is important to keep in mind other ways of achieving precooling (e.g., cold drinks, cooling vest, cooling packs) since previous research postulated that combination of techniques had a significantly larger effect on exercise performance than individual cooling techniques (Bongers et al., 2014; Ross et al., 2013; Tyler et al., 2013; Wegmann et al., 2012).

### Post-exercise

As a result of the demands and load associated with training and competition, and the fact that recovery times are often limited, a central concern for coaches and athletes themselves is to minimize fatigue by enabling adequate recovery as quickly as possible.

Due to its popularity, numerous studies have analyzed the response to or effects of hydrotherapy as a recovery strategy designed to counteract the negative effects of exercise. Some studies have also demonstrated an acute positive effect of cold water immersion on performance, both individual (Vaile et al., 2008) and team sports (Delextrat et al., 2014; King and Duffield, 2009; Versey et al., 2013). It should be noted, however, that some studies have failed to corroborate these effects (Bahnert et al., 2013; Buchheit et al., 2010; Parouty et al., 2010).

The reported benefits of cryotherapy (cold and cool water immersion) include an improvement in perceived recovery and parasympathetic cardiac activity (Al Haddad et al., 2010; Bahnert et al., 2013; Buchheit et al., 2010; Buchheit et al., 2009; Higgins et al., 2012; Ingram et al., 2009; King and Duffield, 2009; Parouty et al., 2010; Stanley et al., 2012; Versey et al., 2011). Cryotherapy may also help to reduce muscle damage and soreness by altering the tissue temperature and improving the blood flow. Its known analgesic effect (it reduces the level of perceived pain) is due to slower nerve conduction velocities and reduced excitability and neural transmission (Bieuzen et al., 2013). Alongside the effect of hydrostatic pressure (see section 4 above) this produces an increase in substrate transport and cardiac output, as well as a reduction in peripheral resistance and the volume of extracellular fluid, via intracellular and intravascular osmotic gradients (Wilcock et al., 2006a). A number of recent studies have reviewed the benefits of using cold water immersion to prevent and treat muscle soreness after exercise (Bleakley et al., 2012; Leeder et al., 2012; Torres et al., 2012; White and Wells, 2013). These reviews conclude that cold water therapy reduces and delays the onset of muscle soreness in comparison with the effects of both passive (rest) and active recovery (low intensity exercise), although the outcomes are not clearly superior to those achieved with massage (Torres et al., 2012). Several studies have also reported a decrease in skin, muscle and core temperature with cold water immersion, which ultimately may benefit recovery (Peiffer et al., 2009; Robey et al., 2014; White and Wells, 2013). In one of the more recent studies, Robey et al. (2013) examined the influence of evening post exercise cold water immersion on subsequent sleep. Results showed that cold water immersion caused a decrease in core temperature (∼2.2°C) until 2.5 h post exercise and there was a 90 min period (3.4–4.5 h post exercise) where core temperature decreased (∼0.3°C). Despite a rapid decrease in core and skin temperatures (compared to exercise alone and control group –no exercise or CWI–), the use of cold water immersion did not influence any aspect of subsequent sleep quality or quantity.

A recent systematic review and meta-analysis by Biezuen et al. (2013) examined the effects that the use of contrast baths (alternating cold and warm water) may have on an athlete’s recovery and muscle soreness following exercise. Contrast water therapy is currently offered to athletes as an equally effective alternative to cryotherapy. It is suggested that it reduces edema and stimulates circulation by alternating peripheral vasoconstriction and vasodilation (Higgins and Kaminski, 1998) or, as in case of cryotherapy, by altering tissue temperature and reducing the blood flow; it is also said to reduce muscle spasm and inflammation, to alleviate and delay the onset of muscle soreness, and to improve the range of motion. In their review, Bieuzen et al. (2013) compared the outcomes of contrast water therapy with those achieved by cold water immersion (cryotherapy), warm water immersion, active recovery (low intensity exercise), compression, and stretching. They concluded that the use of contrast therapy produced significantly greater improvements in muscle soreness recovery compared with no intervention/rest.

With regard to the use of warm water immersion, the literature is scarce and provides limited evidence in terms of outcomes. The effects of heat therapy via warm water are mainly superficial (subcutaneous and cutaneous tissue <2 cm). The principal benefits attributed to warm water baths are an increased blood flow, greater muscle elasticity, and an enhanced range of motion, as well as an analgesic effect and reduced muscle spasm. However, warm water immersion is not recommended as a recovery technique for athletes as it appears to have a detrimental effect on performance (Versey et al., 2013). Note, also, that its use is especially contraindicated in athletes with acute lesions, edema, vascular disease, wounds, or infections (Wilcock et al., 2006a).

The general conclusion would seem to be that contrast baths and cryotherapy are the most effective recovery strategies, at least in comparison with warm water or thermo-neutral immersion (which appear to have no benefits in this regard). However, the use of other popular recovery techniques such as compression, active recovery, or stretching should not be ruled out (Bieuzen et al., 2013; Versey et al., 2013). Importantly, all the reviews in this area highlight a lack of methodological quality or clarity among studies on this topic. Therefore, no definitive conclusions can be drawn with regard to the ideal protocol that should be followed (with any form of hydrotherapy) in order to optimize an athlete’s recovery. Generally speaking, immersions in water at 10–15 °C for between 5 and 15 min (either in a single immersion or several short periods) seem to be the most effective way of speeding up the recovery process. When using contrast baths the recommendation would be to alternate cold (8–15 °C) and warm (>36 °C) water immersions for ∼1–2 min, respectively, with a total duration of around 15 min (Versey et al., 2013).

As a general conclusion, although there is a large body of research conducted to evaluate the use of water immersion for exercise recovery, further studies are needed to confirm the optimal water temperatures, immersion durations, depths timing post-exercise and timing before subsequent exercise, and also to analyze the long-term effect of chronic use of hydrotherapy in subsequent training sessions.
